# Spatio-temporal distribution of human lifespan in China

**DOI:** 10.1038/srep13844

**Published:** 2015-09-08

**Authors:** Shaobin Wang, Kunli Luo, Yonglin Liu

**Affiliations:** 1 Institute of Geographic Sciences and Natural Resources Research, Chinese Academy of Sciences, Beijing 100101, China; 2 University of Chinese Academy of Sciences, Beijing 100049, China; 3 College of geography and Tourism, Chongqing Normal University, Chongqing 400047, China

## Abstract

Based on the data of latest three Chinese population censuses (1990–2010), four lifespan indicators were calculated: centenarians per one hundred thousand inhabitants (CH); longevity index (LI); the percentage of the population aged at least 80 years (ultra-octogenarian index, UOI) and life expectancy at birth (LEB). The spatio-temporal distributions of data at Chinese county level show that high-longevity areas (high values of CH and LI) and low-longevity areas (low CH and LI values) both exhibit clear non-uniformity of spatial distribution and relative immobility through time. Contrarily, the distribution of UOI and LEB shows a decline from the east to the west. The spatial autocorrelation analyses indicate less spatial dependency and several discontinuous clusters regions of high-CH and LI areas. The factors of temperature, topography and wet/dry climate lack of significant influence on CH and LI. It can be inferred that, in addition to genetic factor and living custom, some unique and long-term environmental effects may be related with high or low values of CH and LI.

Human lifespan is a mysterious issue, and long lived members of a population are of considerable interest. The long human life span has always been a symbol of health in most societies, especially for those who live longer than one hundred years old, who may either delay or avoid life-threatening illnesses^1^. The proportion of centenarians and longevity index (LI) have been applied to investigate in several “longevity islands”^2^,^3^,^4^. Furthermore, China, with the largest population in the world, is recognized with the regional phenomenon of longevity^5^. Several studies have identified the distribution of longevity population based on one or two Chinese population censuses at provincial level^6^,^7^,^8^,^9^,^10^,^11^. Those researches indicated an uneven-distributed pattern of longevity population regions which shows a great difference between provinces or even counties.

With development of China’s economy and society, more detailed and accurate demographic data at all age groups have been obtained from Chinese national population censuses. Moreover, for the household registrations system restricting the population mobility, the Chinese elderly residents always live most of their lives in there hometowns. In addition, China has distinct natural geographic differentiation (e.g. topography, wet/dry and climate), complicated geological environment and geologic history. The natural geographic factors that related with human longevity in China at provincial level were involved by some studies^10^,^12^, whereas non-zonal factors such as geological environment have not even been considered.

The spatio-temporal distribution of human lifespan, a thought-provoking scientific issue of public concern, will provide a useful perspective on human health and environment. However, there has no systematic analysis of the distribution and variation of human life span in China with high resolution (at the county level). It is still unclear about the patterns of temporal and spatial distribution of human lifespan in China at county-level, especially for the areas with extremely high or low values. In this paper, based on specific data from Chinese national censuses, we calculated and integrated the four indicators as the lifespan index: centenarians per one hundred thousand inhabitants (CH); longevity index (LI); the percentage of the population aged at least eighty years (ultra-octogenarian index, UOI) and life expectancy at birth (LEB). The first three are individual indexes and the last is demographic index, thus, the distribution of human life span can be investigated both individually and statistically.

In this paper, we mainly identify the spatial-temporal distribution patterns of those indicators of more than two thousand Chinese counties during the past 30 years. Based on correlation analysis between lifespan indicators and physical geographic factors, as well as spatial autocorrelation (SA) analysis, the possible distribution mechanism and potential implications of nature environmental health issues were evaluated as well. Those that related to distribution of high or low life span indicators will be beneficial to the understanding of the relation between human lifespan and physical geography factors. The health information of international significance from the distribution pattern and influence degree of natural geographical environment could enable policy-makers, medical workers and researchers to be more effective.

## Results

### County-level distributions of lifespan indicators in China

**Figure 1:**
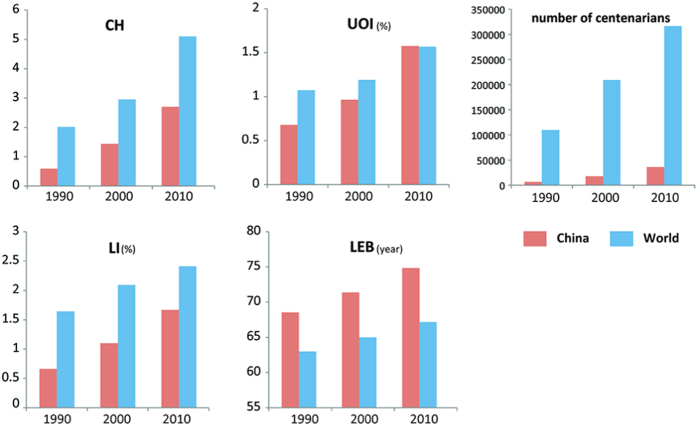
Variation in CH, LI, UOI and LEB values in China and the world (Data sources: UN World Population Prospects 2010).

The world average CH-value is 5.1 in 2010^13^. Since the 1950s, the number of centenarians in industrialized countries doubled every ten years approximately, and from the early 1950s to the late 1980s, the average annual growth rate of centenarians was about 7% in these countries^14^. The national population censuses in China reveal a similar increasing trend of the indicators such as CH, LI, UOI and LEB (Fig. 1). The indicators such as UOI and LEB of China are close to or even exceed the world average level. In contrary, the CH and LI are obviously lower than the world average values (Fig. 1).

**Figure 2:**
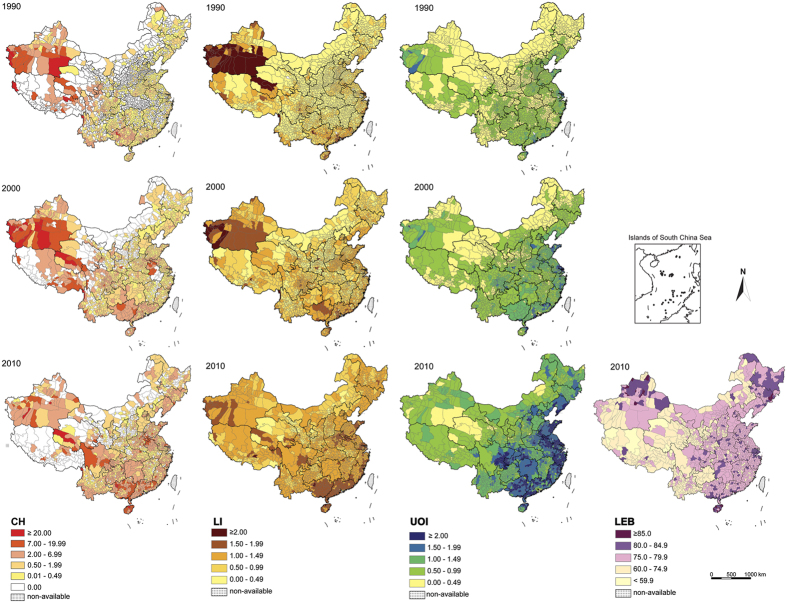
Distribution and variation of CH, LI, UOI and LEB in mainland China (1990–2010) at county level. The maps were created using Arc GIS Geographic Information Systems software version 10.0 (Environmental Systems Research Institute Inc, Redlands, Calif).

**Figure 3:**
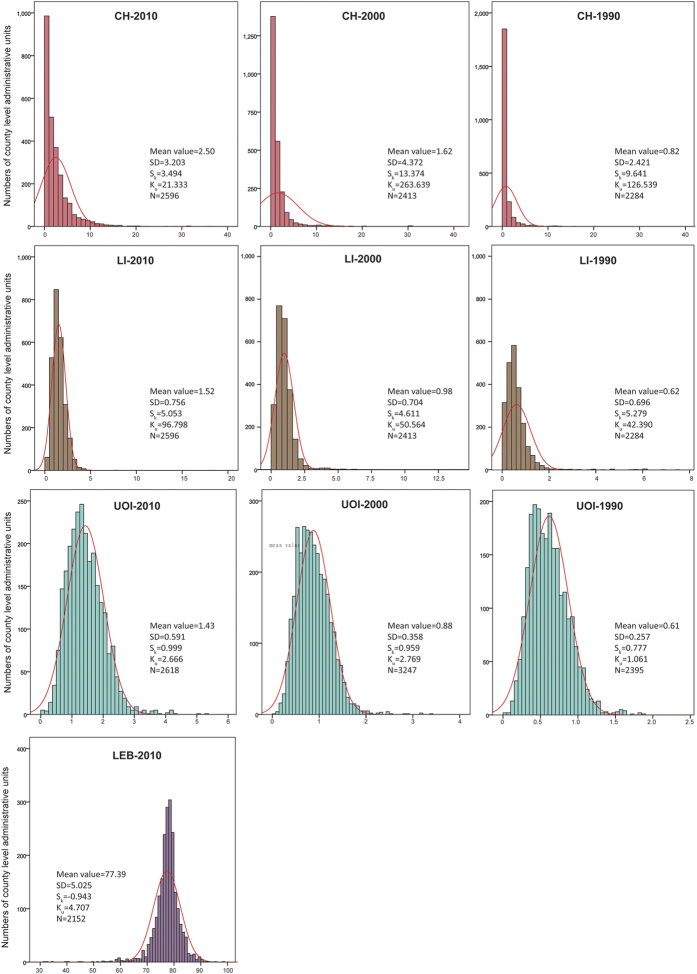
Frequency histograms and fitting normal distribution curves of lifespan indicators in 1990, 2000 and 2010 at county level in China.

Based on the latest three national population censuses in China at county-level, the lifespan indicators in south China were persistently slightly higher than those in north China, except LEB in 2010 (Table 1). The values of lifespan indicators in Tibetan area were obviously lower than both of north and south China in view of the census in 2010 (for data quality see methods part). Obvious geographical distribution of UOI and LEB shows a clear decline from the east to the west rather than difference between North and South China (Fig. 2). CH and LI values exhibit scattering and cluster distribution (Fig 2). Distribution of CH and LI is characterized by skewness of the distribution, while distribution of UOI and LEB approximately exhibits a normal distribution (Fig 3). In addition, these distribution patterns changed little across time.

### Four regions with high-centenarian-ratio in China

Although the total number of centenarians grows form 1990 to 2010, high-CH areas show relatively stable feature through time succession. From those data of centenarians at county-level, high-CH areas were mainly concentrated in the following four regions (Fig. 2):

#### Lingnan Region

This area mainly refers to Guangxi, Guangdong and Hainan Provinces in natural geographical framework. In particular, Hechi Prefecture in Guangxi Province and north part of Hainan Island show the highest CH values in China.

#### Sichuan Basin

Distribution of centenarians is mainly in the central and western of Sichuan Basin.

#### Huanghuai Region (“SHA” region)

It is remarkable that the CH values in the junction of southwestern Shandong Province, eastern Henan Province and northern Anhui Province are much higher than adjacent areas, which form a distinct longevity region in North China Plain.

#### South of Xinjiang Autonomous Region

This area is a traditional longevity region in China. Although the first three census data had questionable accuracy, this area still has obvious phenomenon of regional longevity based on more reliable census data after the census in 1990.

### Spatial autocorrelation of lifespan indicators

#### Global spatial autocorrelation (Moran’s I)

To compare the spatial autocorrelations of different lifespan indicators, global Moran’ s *I* statistics are calculated at county-level in China (1990–2010). Table 2 lists global spatial autocorrelations and their significant of Moran’s *I* with *Z*-test. The global Moran’s *I* from 1990 to 2010 indicates difference significant positive spatial autocorrelation of lifespan indicators as UOI > LI > CH. A significant positive autocorrelation of UOI indicates the clustering both of high-value and low-value, and weak or non- significant autocorrelation of LI and CH may be attributed to the trend of spatial stochastic distribution. The difference of Moran’s *I* between different lifespan indicators (UOI > LI > CH) may imply that spatial dependencies are weakened with the age growth of the elderly population to longevous population. The ascending global Moran’s *I* indicates an upward trend of spatial association from 1990 to 2010 (Table 2). In addition, LEB at country level in 2010 shows a much lower positive autocorrelation than previous studies at provincial level^15^. It may be explained by the scattering distribution of cities with high values of LEB among more counties with low level of LEB.

#### Local spatial autocorrelation (Local Moran’s I)

**Figure 4:**
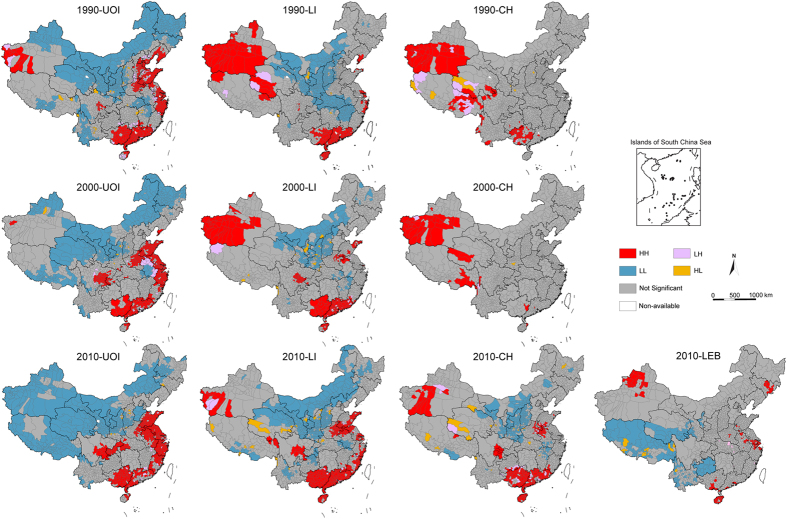
Map of local spatial autocorrelation of lifespan indicators (UOI, LI and CH) in China in 1990, 2000 and 2010. The maps show significant areas with *p* < 0.05 as red, blue, purple and yellow, and no significant areas as grey. High-high (HH) and low-low (LL) = spatial clusters; High-low (HL) and low-high (LH) = spatial outliers. The maps were created using Arc GIS Geographic Information Systems software version 10.0 (Environmental Systems Research Institute Inc, Redlands, Calif).

Figure 4 shows the areas with significant locations color-coded by different types of spatial autocorrelation (Local Moran’s *I*) of four lifespan indicators, respectively. HH districts of UOI are mostly located in eastern coastal areas and inland Sichuan, Chongqing and western Hubei Provinces, whereas LL districts are mainly in northwestern China, Tibet, Inner Mongolia and Shanxi Provinces (Fig 4). The HH and LL areas of LI are smaller than UOI, whereas the south Xinjiang Autonomous Region is recognized as HH districts (Fig 4). HH districts of CH are composed by four obvious clusters: south China area (Guangxi, Guangdong and Hainan Provinces), Sichuan Basin, Huanghuai district and southern Xinjiang Autonomous Region, which is consistent with the four regions with high-centenarian-ratio mentioned above. Whereas the LL districts of CH exhibit an obvious cluster area with relative immobility through time as well, which includes Shanxi, Shaanxi, Gansu and Inner Mongolia Provinces (Fig 4). Furthermore, LEB at country level in 2010 shows much smaller HH districts than other indexes (Fig 4), which can be explained by the scattering distribution of cities with high LEB values among more counties with lower LEB level.

From the maps depicted above, different local spatial autocorrelation of those lifespan indexes can be identified. Local SA of UOI show an east-west differentiation, whereas the local SA of CH and LI exhibits only several discontinuous clusters areas. Compared with large distribution areas of autocorrelation of UOI, the HH-type areas of CH are quite small and discontinuous in geographical distribution. Most of the counties show no significant spatial autocorrelation of CH.

### Spearman’s rank correlation coefficients between lifespan indicators and zonal physical geographic factors

**Figure 5:**
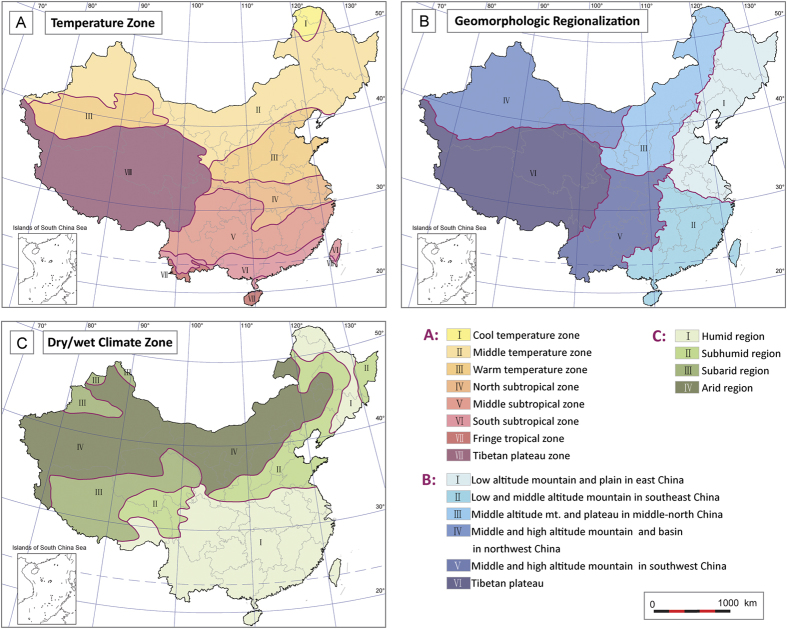
Zonal distribution of natural geographic factors in China (schematic maps modified from references 16, 17 ). (**A**) Temperature zone regionalization; (**B**) Geomorphologic regionalization; (**C**) Dry/wet climate regionalization. The maps were created using Arc GIS Geographic Information Systems software version 10.0 (Environmental Systems Research Institute Inc, Redlands, Calif).

Some natural environmental factors exhibit a pattern of zonal distribution such as solar radiation, temperature, precipitation and so on. The identification of the differentiation and similarities among different areas and regionalization of these physical geographic factors in China had been conducted and mapped (Fig 5)^16^,^17^. The relation between the changes of these parameters and lifespan indicators can be detected by using the Spearman’s rank correlation coefficients. In this paper, each variable of physical geographic factors at county level in China is ranked from lowest to highest. Then Spearman’s coefficients between these variables and lifespan indicators are derived. We computed Spearman’s rank correlation coefficient and its 99% confidence intervals to assess the relationship between variables of interest (Table 3).

The results show that most of the correlation coefficients are quite low, though by significance test, due to large sample (Table 3). The correlation coefficients greater than 0.4 will be considered as moderately correlated, whereas those lower than 0.4 as weakly related^18^. The results show that regionalization of temperature, topography (elevation) and wet/dry climate have relatively weak correlation coefficients with CH and LI, which do not suggest very significant correlation (Table 3). Whereas, moderate negative correlation can be observed between geomorphologic and UOI (*r* = −0.471), and LEB (*r* = −0.542), and between wet/dry climate factor and UOI (*r* = −0.458) (Table 3).

## Discussion

Human longevity is not a common phenomenon, moreover, regions with high ratio of longevous population are quite rare as well. Sardinia, Okinawa, Ikaria and Costa Rica are characterized by reported high ratio of centenarians^2^,^4^,^19^. The longevity in those areas, including isolated islands and coastal environments is even puzzling. In this paper, we explored the spatial distribution of the high and low-longevity areas in China which exhibits clear non-uniformity of spatial distribution and relative immobility through time. These distribution patterns have persisted despite regional population restructuring, social and economic development.

The persistent geographic pattern of population mortality rate in U.S. was illustrated. Distribution maps of county mortality rates showed persistent features both of temporal and spatial which exhibited clustering of high and low mortality rates^20^. The counties with high/low mortality rates in U.S. experienced different socioeconomic development such as population outmigration, medical conditions, and socioeconomic policies. Our results underscore that high-longevity areas and low-longevity areas in China both show clear non-uniformity of spatial distribution and relative immobility through time. Although this paper from the lifespan perspective is different with the mortality rates distribution of U.S., they both suggest that long-term environmental factors may play an important role that has potential influence on human lifespan.

It is widely recognized that the human lifespan is related with various factors such as living environment, health care condition, dietary and nutrition, heredity and psychological factors, etc.^5^,^21^,^22^,^23^,^24^,^25^,^26^,^27^. Different longevity indexes exhibit different relations to economic level (per capita GDP) in China, that is, the distributions of UOI and LEB are significantly affected by economic conditions, but in contrary, the values of CH and LI lack any significant correlation with economic level. So it is indicated that economic conditions may have limited impact on human lifespan, especially for those who live longer than 90 years old^28^. This finding may related with the distribution patterns of longevity indicators that showed both temporal and spatial persistent feature despite regional social-economic development.

Some researchers identified that cold environment was more conducive to human or animal’s lifespan extension^29^,^30^,^31^,^32^,^33^. And some research showed that low latitude area is more conducive for human lifespan extension^10^,^12^. But the results in this paper do not indicate a very significant correlation between temperate, topography (elevation) and wet/dry climate regionalization and distribution of CH and LI in China based on high resolution data at the county level. High-CH and LI areas are mainly distributed in several discontinuous clusters areas which is not consistent with latitudial zonality of climatic regionalization and approximate longitudinal zonality of topography and wet/dry climate. Whereas, moderate negative correlation can be observed between geomorphologic and UOI, and LEB, and between wet/dry climate factor and UOI, which is interpreted by east-west differentiation of UOI and LEB that is consistent with the longitudinal zonality of these factors. This founding does not support the view that climate, topography, geomorphology, longitude, and even sunshine are the dominant determining factors of longevity (high CH and LI values)^12^,^34^. The results revealed in this paper may indicate more complex environmental factors rather than geographic influence that related with the differentiation of lifespan and high-longevity areas in China.

Furthermore, it can be inferred that, in addition to genetic factor and living custom, some unique and long-term effects of certain tectonic background may have impacts on the environment related with high or low values of CH and LI. These findings may imply the importance of further studies on “health and environment” issue. Compared with human individual life span and even the history of human evolution, geological background is a long-term effect and relatively stable as well. The geological bodies exhibit a pattern of non-zonal distribution in the earth surface, and the physical and chemical properties of bedrock and sediment, which are the source of the elemental composition of soil and water in the area, may potentially impact on human lifespan. To elucidate the commonalities of the geological background in high- and low- longevity areas, further comprehensive studies will be needed.

## Methods

### Lifespan indicators

The terms life span and longevity in this paper are mainly two terminologies interchangeable, both of which refer to the period of human from birth to death. The word “longevity” often refers to long lived members especially for those who live more than ninety years old^35^, while “life expectancy” is always defined as the mean number of years remaining at a given age until death under specific mortality conditions^36^. The most commonly use of life expectancy is life expectancy at birth (LEB), which refers to a mean length of life since birth until death based on the mortality rates observed at a certain year^37^. LEB is a measure of overall quality of life in a country and summarizes the mortality at all ages. Because current global average LEB is less than a hundred, the terminology of “centenarians” is associated with longevity invariably^38^,^39^.

The proportion of centenarians is the most direct and convincing index of longevity^2^,^3^,^4^. Thus, in this article, the number of centenarians per one hundred thousand inhabitants (CH) is used to represent the extent of centenarians. Another important indicator used by some researchers is the longevity index (LI), defined as the ratio of the population above 90 years of age over the total population above 65 years of age^35^. The influence of migration and birth rates can be minimized by using the LI than taking the total population as a basis. Therefore, this paper defines population over 90 years old as longevous population indicated by CH and LI, and those areas with high-CH and LI values are considered as high-longevity areas. Conversely, areas low high-CH and LI values are considered as low-longevity areas. We also use the percentage of the population aged at least 80 years (ultra-octogenarian index, UOI) as a reference indicator.

### Data sources

Population data at the provincial level (i.e. provinces, province-level autonomous regions and municipalities) are from Chinese national population censuses^40^,^41^,^42^,^43^. The population data in 1990 at county-level were from China National Science and Technology Infrastructure (NSTI program): Data sharing Infrastructure of Earth System Science (www.geodata.cn). The population data in 2000 and 2010 at county-level are from provincial data of fifth and sixth national population censuses in China. There are no data at county-level in Liaoning, Hebei, Hunan, Guangxi and Guizhou provinces in 2000 and in Liaoning, Hebei, Hunan and Sichuan provinces in 2010, which are instead of prefecture-level data. There is not any information about longevous population in Anhui in 2000, so we used 1% population sample survey data in 2005 instead^44^. The data of dead population in 2010 at county-level or prefecture-level are from sixth national population censuses in China.

### Data quality assessments and calculation

The accuracy of the first two national population censuses in 1953 and 1964 was questioned by some researchers^45^,^46^, but these data still have an important value for reference. China’s first high-quality population census was launched in 1982, with the assistance of the United Nations Population Fund. The reliability of the data has been recognized by some scholars and organizations^46^,^47^,^48^,^49^. However, the accuracy of longevity population data of Xinjiang Autonomous Region in 1953, 1964 and 1982, as well as other ethnic minority areas such as Tibetan area, is quite questionable, especially for the centenarian numbers. The gap between verified ages and self-reported ones by “centenarians” in those areas was questioned by some researches^11^,^50^,^51^,^52^ which does not meet the requirements as a factual basis to study. Thus, data of Tibetan area in 1990 are only for reference in this article.

Fortunately, the quality of Chinese national census in 2000 and 2010 is much better than previous censuses^53^,^54^. The under-enumeration rate of sixth census is even lower than those in recent censuses by some developed countries^54^.

We calculated the three assessment indicators (CH, LI and UOI) for each counties in 1990, 2000 and 2010. The calculation of LBE is based on the method of life table^55^.

### Spatial autocorrelation analyses

To evaluate the spatial pattern of lifespan indicators, we examine the global and local spatial autocorrelation by using the Moran’s index (Moran’s *I*) and Local Moran’s index (Local Moran’s *I*) calculated by Arc GIS 10.

Moran’s *I* can be expressed as follows^56^:


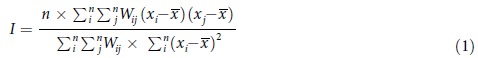


In Eq. (1), *n* is the number of spatial units indexed by *i* and *j*; *x* is the variable of interest; *xi* and *x_j_* are the values of the observed variable at sites *i* and *j*; 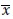 is the mean of *x*; the weights *W*_*ij*_ are written in a (*n* × *n*) weight matrix; The weight matrix depicts the relation between an element and its surrounding elements. Weight can be based on contiguity relations or distance. The value of Moran’s *I* generally range from −1 to +1. Negative (positive) values indicate negative (positive) spatial autocorrelation. For statistical hypothesis testing, values of Moran’s *I* can be tested based on their Z-scores, that is, |*Z*| > 1.96 or |*Z*| > 2.54 indicate spatial autocorrelation that is significant at the 0.05 or 0.01 confidence level.

The Moran’s *I* only reveal the presence or absence of spatial autocorrelation generally. The local spatial autocorrelation can measure the spatial distribution patterning of variate values in a locality^56^. The spatial distribution of local SA can be expressed by Local Moran’s *I*^57^. Specifically, local SA analyzes the extent to the value of a variable *x* at a certain location is related to that variable at its neighboring areas.


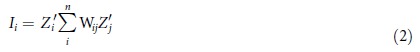


In Eq. (2), *Z*_i_’ and *Z*_j_’ are the original variables of *x*_i_ and *x*_j_ in standardized forms, respectively; and *W*_*ij*_ is the spatial weight matrix. A positive/negative value for Local Moran’s *I* indicate that a feature has neighboring features with similarly high or low attribute values/ dissimilar values. Map of spatial autocorrelation distinguishes between (a statistically significant at 0.05 level) cluster of high values (HH), cluster of low values (LL), outlier in which a high value is surrounded primarily by low values (HL), and outlier in which a low value is surrounded primarily by high values (LH).

### Spearman’s rank correlation coefficients

Spearman’s rank correlation coefficient is a nonparametric method to assess how well the relationship between two independent variables without normality assumption of the raw data^58^. It operates on the ranks of the data and each variable is ranked separately from lowest to highest (e.g. 1, 2, 3, etc.) and the difference between ranks for each data pair is recorded. This method can be useful even when the actual values of items are unknown. The disadvantage is that there is a loss of information when the data are converted to ranks. The Spearman’s rank correlation coefficient is calculated according to the following equation:


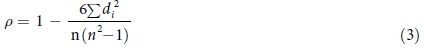


In Eq. (3), *d*_*i*_ = *x*_*i*_*–y*_*i*_, _*i*_s the difference between the ranks of two items; *n* is the number of observations. Calculated Spearman’s rank correlation coefficient (*ρ*) is between −1 (a perfect negative correlation) and +1 (a perfect positive correlation).

This method is suitable for estimating the relationship between different type of physical geographic factors (at different ranks) and lifespan indicators. We calculated the Spearman’s rank correlation coefficients by SPSS version 19.0.

## Additional Information

**How to cite this article**: Wang, S. *et al.* Spatio-temporal distribution of human lifespan in China. *Sci. Rep.* **5**, 13844; doi: 10.1038/srep13844 (2015).

## Figures and Tables

**Table 1:** Comparison of lifespan indicators between North, South China and Tibetan Area (natural geographical units)*.

**Lifespan indicators**		**South China**	**North China**	**Tibetan Area**
LEB	2010	77.31	78.21	71.12
UOI	2010	1.60	1.31	0.74
	2000	0.97	0.77	0.56
	1990	0.65	0.59	0.45
LI	2010	1.71	1.33	1.18
	2000	1.03	0.94	0.80
	1990	0.63	0.59	0.63
CH	2010	2.93	2.09	1.35
	2000	1.44	1.69	2.41
	1990	0.76	0.69	2.08
*Number of counties*		*1697*	*1345*	*153*

^*^The north China and South China is divided by Qinling Mountain- Huaihe river.

**Table 2:** Global spatial autocorrelations of lifespan indicators form 1990 to 2010*.

	**Year**	**Moran’s** ***I***	***Z***	***P-*****value**
UOI	1990	0.373	159.730	<0.0001
	2000	0.407	91.343	<0.0001
	2010	0.536	123.713	<0.0001
LI	1990	0.217	93.787	<0.0001
	2000	0.214	48.348	<0.0001
	2010	0.470	108.355	<0.0001
CH	1990	0.070	30.834	<0.0001
	2000	0.080	19.525	<0.0001
	2010	0.245	60.204	<0.0001
LEB	2010	0.104	23.476	<0.0001

^*^Conceptulization of spatial relationships: Inverse distance; Distance method: Euclidean distance.

**Table 3:** Spearman correlation matrix between physical geographic factors and lifespan indicators in China (1990, 2000, 2010).

**Physical Geographic Factor**	**CH**			**LI**			**UOI**			**LEB**
	**1990**	**2000**	**2010**	**1990**	**2000**	**2010**	**1990**	**2000**	**2010**	**2010**
*ρ* (Temperature zone)	0.242^**^	0.205^**^	0.212^**^	0.263^**^	0.220^**^	0.333^**^	0.200^**^	0.259^**^	0.161^**^	−0.189^**^
*ρ* (Geomorphologic)	0.062^**^	0.030	−0.236^**^	−0.130^**^	−0.170^**^	−0.346^**^	−0.316^**^	−0.279^**^	−0.471^**^	−0.542^**^
*ρ* (Dry/wet Climate)	−0.184^**^	−0.090^**^	−0.263^**^	−0.163^**^	−0.152^**^	−0.327^**^	−0.230^**^	−0.382^**^	−0.458^**^	−0.112^**^
*N (number of Counties)*	*2284*	*2413*	*2596*	*2284*	*2413*	*2596*	*2284*	*2413*	*2596*	*2573*
